# Two chromatographic methods for analyzing paracetamol in spiked human plasma with its toxic metabolite, *N*-acetyl parabenzoquinone imine and its antidote, *N*-acetyl-l-cysteine

**DOI:** 10.1038/s41598-025-86070-3

**Published:** 2025-02-03

**Authors:** Omar M El-Abassy, Michael Gamal Fawzy, Ebraam B. Kamel

**Affiliations:** Pharmaceutical Chemistry Department, Faculty of Pharmacy, Egyptian Russian University, Badr City, Cairo, 11829 Egypt

**Keywords:** Detoxification, *N*-acetyl para benzoquinone imine, Paracetamol, Acetylcysteine, Chromatography, Plasma analysis, Blue applicability grade index, Analytical chemistry, Green chemistry

## Abstract

Acetaminophen, also known as paracetamol (APAP), is a highly utilized pharmaceutical agent on a global scale, particularly in the field of pediatrics. Regrettably, an overdose of APAP, resulting from the predominant oxidation, has the potential to trigger acute liver injury. The study’s goal was to find an easy, accurate, and selective way to measure APAP, *N*-acetyl para benzoquinone imine (NAPQI) (an APAP metabolite that is harmful), and *N*-acetyl-l-cysteine (NAC) (an antidote). Two different chromatographic methods were used. The HPTLC method, which used silica gel 60 F_254_ as a stationary phase and a developing liquid made up of methanol, ethyl acetate, and glacial acetic acid (8:2:0.2, v/v/v) and a UV detection at 254 nm. The HPLC method was developed using a mobile phase consisting of water, methanol, and formic acid in a proportion of (70:30:0.15, v/v/v). The stationary phase used in the approach was a C_18_ column. Analytes quantification was established utilizing a UV detector operating at a wavelength of 254 nm. The present methods make it possible to measure the amount of APAP in plasma samples. When it comes to pharmacokinetics or medication levels in children’s plasma, for example, this may be also very helpful. The current methods can quantify NAPQI, which is helpful in figuring out drug concentrations in individuals with APAP intoxication diagnoses. Additionally, the current approaches can estimate NAC as an antidote; as a result, this study is a complete study because it can analyse drug, toxic metabolite, and antidote in one analytical run. Using the innovative blue applicability grade index software, which measures the practicality of procedures, both methodologies were compared with a reported methods. Additionally, the achievement of the eco-friendliness profile of the designed procedures was assessed. Both techniques passed the ICH validation tests.

## Introduction

**Fig. 1:**
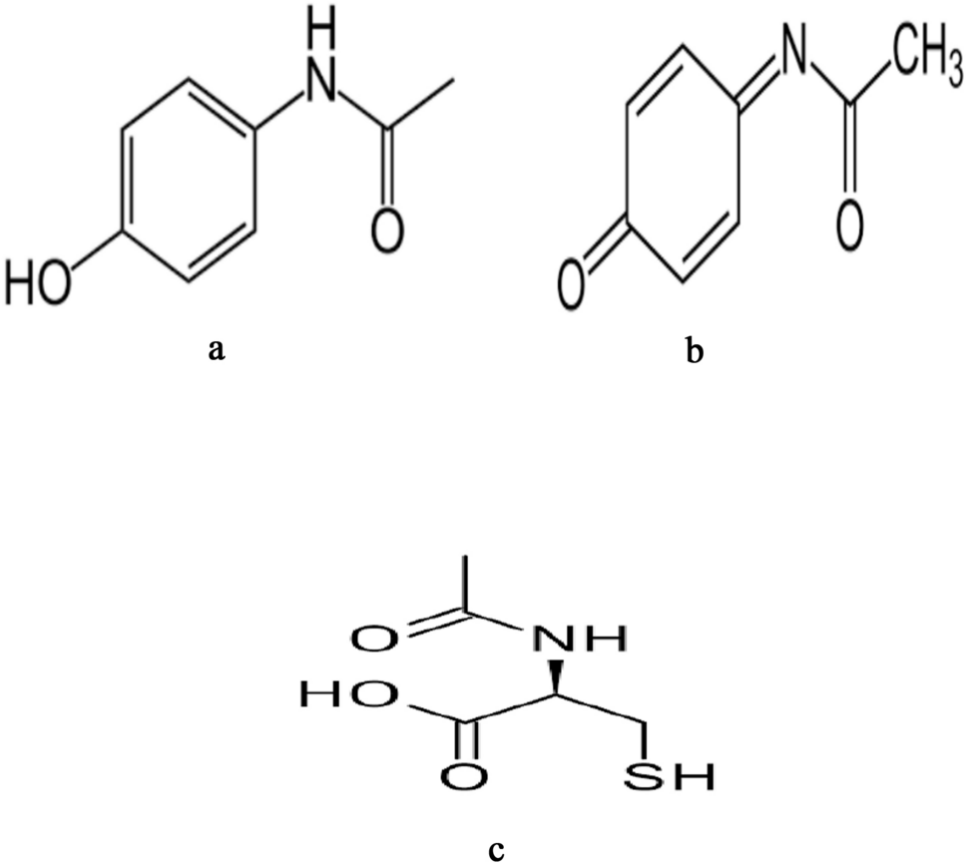
Chemical structures of APAP (**a**), NAPQI (**b**) and NAC (**c**).

APAP (Fig. 1a) is a prominent pharmaceutical agent commonly employed for the management of both fever and pain^1^. It has worldwide popularity and is extensively utilized. The administration of APAP within the recommended dosage ranges has been shown to exhibit a favorable safety profile. APAP is frequently prescribed for pediatric patients due to its well-established safety profile when administered within the recommended therapeutic dosages. Roughly 50 million adults in America regularly consume products that contain APAP on a weekly basis. Nevertheless, the excessive consumption of APAP can lead to substantial liver damage^2^. Acetaminophen (APAP) toxicity generally becomes apparent within the initial 24-h period following administration. It is characterized by general symptoms including nausea, vomiting, abdominal pain, and weakness^3^. These signs have the potential to resolve within a 24-h timeframe. Nonetheless, 3–4 days after medication delivery, signs of hepatic injury, like jaundice, liver discomfort, and an enlarged liver, may manifest^4–6^.

Approximately 5–10% of APAP undergoes hepatic metabolism by cytochrome CYP 2E1, as a consequence, a highly reactive and harmful metabolite known as *N*-acetyl-para-benzoquinone imine (NAPQI), Fig. 1b is produced. When used in therapeutic amounts, the toxic metabolite is detoxified by attaching to the sulfhydryl group of glutathione (GSH). This creates non-toxic cysteine and mercapturic acid sections. These moieties are subsequently excreted in the urine^7,8^.

In cases of APAP overdose, the bonding of glutathione (GSH) with the NAPQI metabolite results in GSH depletion. This depletion causes NAPQI to covalently bind to macromolecules, particularly proteins, which subsequently leads to malfunction of the mitochondria. As a consequence, the formation of peroxynitrite and reactive oxygen species (ROS) occurs^9,10^. Hence, necrotic liver and the opening of the mitochondrial membrane permeability transition (MPT) hole are caused by ROS formation^11,12^. Consequently, a viable approach to mitigating liver damage involves the restoration of GSH levels within hepatocytes through the utilization of GSH prodrugs^13^. NAC (Fig. 1c) is responsible for the replenishment and preservation of hepatic GSH reserves. It achieves this by acquiring the cysteine amino acid, which serves as the substrate for detoxifying risky metabolites of APAP, including NAPQI^14^.

There have been many descriptions of techniques for APAP metabolism analysis. Their results are crucial for pharmacodynamics, pharmacokinetic, and toxicological research. like LC–MS/MS^15–21^, LC-UV^22–26^and capillary electrophoresis^27–29^.

So far, there have been no known analytical techniques that can simultaneously determine APAP, NAPQI, and NAC as an antidote. This presents an excellent opportunity to conduct an analysis using two straightforward chromatographic methods.

The current HPLC technique is characterized by its emphasis on speed and simplicity. This is achieved through short analytical run for the HPLC technique, typically lasting only 5 min. that allows the use of minimal solvent quantities. The HPTLC technique is a trustworthy, effective, and economical approach to the separation and quantification of a wide range of analytes.

Lately, there has been a noticeable push to incorporate ideas from green chemistry and green analytical chemistry (GAC). As a result, several criteria were developed to evaluate the ecological soundness and friendliness of the analytical approaches, with the ultimate goal being to ascertain their greenness^30–34^. The Green Analytical Procedure Index (GAPI) and Analytical GREENNESS metric approach (AGREE) were the two metric tools used in the current study to assess the exceptional greenness of the suggested procedure^35,36^. Besides the level of applicability of the approaches were demonstrated by the BAGI tool.

Two novel methodologies that use chromatography were subsequently developed and validated for the quantification of the aforementioned analytes. In this article, we presented two chromatographic techniques that can be employed for simultaneous determination of the current analytes, with the aim of achieving cost-effectiveness, time efficiency, green, applicability and improved suitability for routine analysis. In the realm of pharmaceutical analysis, the chromatographic procedures that have recently been developed are both unique and extremely significant. In line with ICH standards, the present procedures were validated^37^.

## Experimental

### The apparatus and Instruments

#### HPTLC method

The HPTLC instrument was used in conjunction with an automatic-sampler (A CAMAG Linomat 5) and a 100-µL CAMAG syringe (Muttenz, Switzerland) for placing the samples on a thinner plate silica gel aluminum paper (20 × 10 cm) and thickness of (0.1–0.2 mm) (Merck, Darmstadt, Germany. For the detection and spots scanning, 3S/N 1302139 CAMAG detector (Muttenz, Switzerland) and the Win CATS^®^ program were both employed.

#### HPLC method

The study used an HPLC unit from Milford, MA, USA. It had a degassing system, an Agilent 996 photodiode array detector (DAD), and an Agilent 2690 HPLC separation that was linked to a quaternary pump. The Empower 3 chromatography software system was used to process and set up the data. Separation was done with a Zorbax^®^ SB C_18_ column (4.6 × 250 mm, 5 µm). A pH meter from Essex, UK, was used to measure the pH. A Soniclean 120 T ultrasonic cleaner (Thebarton, South Australia) was used to clean the instruments.

### Materials and methods

#### Pure samples

Amoun Pharmaceuticals (Cairo, Egypt) kindly gave us APAP with a certified purity of 99.88%. EVA Pharma (Cairo, Egypt) kindly gave away NAC with a certified purity of 99.88%. The NAPQI compound was acquired from Sigma Aldrich (Saint Louis, MO, USA) with a certified purity of 99.99%.

#### Chemicals and reagents

El-Nasr Pharmaceutical Chemicals (Cairo, Egypt), provided a selection of analytical-grade chemicals, including ethyl acetate, acetic acid, and formic acid. Sigma Aldrich (Saint Louis, Missouri, USA) was the supplier of methanol (HPLC grade). We also utilized Otsuka Pharmaceutical (Cairo, Egypt) double-distilled water.

### Methods

#### Chromatographic setup

##### HPTLC method

The separation process is achieved using a stationary phase of silica gel sheets 60 F_254_ HPTLC plates with dimensions of (20 × 10 cm). A suitable portion of the stock solution was automatically dispensed and formed into compact bands. A developing system consisting of a mixture of methanol, ethyl acetate, and glacial acetic acid in a ratio of (8:2:0.2, v/v/v) was utilized. This developing system was then moved to a twin-trough chamber made of glass. After a saturation period of 20 min at room temperature, the plates were meticulously handled to the glass jar and permitted to experience a linear ascending progression of the developing system for a distance of 9.0 cm. Subsequently, we elevated the plates to facilitate air drying. The densitometric measuring scan employed at a wavelength of 254 nm.

##### HPLC method

A mixture of water, methanol, and formic acid in the proportions (70:30:0.15, v/v/v) was used with isocratic elution. The elution was carried out at a flow rate of 1 mL min^−1^. The analysis column utilized in this study was a Zorbax® SB C_18_ (4.6 × 250 mm, 5 μm). Following an ultrasonic degassing process for 15 min, before being utilized, the mobile-phase liquids were passed through a 0.45-m Millipore membrane filter. A precise separation was attained at a wavelength of 254 nm. Prior to the procedure of triplicate injection on the HPLC device, the samples were filtered through a 0.45 µm Millipore membrane filter. An auto-sampler with a volume of 20 μL was used.

#### Linearity

##### HPTLC method

After establishing the stock standard solutions (1 mg mL^−1^), we injected precise dilutions of APAP, NAPQI, and NAC three times as bands employing the HPTLC equipment and the previously instilled chromatographic conditions. The dilutions were within the specified ranges of 0.5–6, 1–7, and 0.3–18 μg band^−1^ for APAP, NAPQI, and NAC respectively. The average peak areas were plotted against the relevant concentrations to construct the calibration curves. The collected scanning profiles were employed for generating the regression equations.

##### HPLC method

From stock solution (1 mg mL^−1^) and working solution (100 μg mL^−1^) we produced and evaluated linearity solutions of APAP, NAPQI, and NAC utilizing the mobile phase as a solvent. The concentrations of these solutions ranged from 1 to 30 μg mL^−1^, 10 to 200 μg mL^−1^, and 3to 100 μg mL^−1^, respectively. Utilizing the established chromatographic criteria, three replicates of the solutions that were created were injected. The concentrations were determined by acquiring chromatograms and then using them to build a calibration curves showing the average peak areas.

#### Laboratory-prepared mixtures of APAP, NAPQI, and NAC

Aliquots of APAP, NAPQI, and NAC were accurately dispensed into three sets of 10-mL volumetric flasks, for HPTLC stock standard solutions was employed while utilizing working standard solutions for HPLC/DAD. The substances were combined and appropriately diluted using methanol for HPTLC and mobile phase for HPLC/DAD, respectively. The mixtures were developed in accordance with the previously mentioned procedures for each respective method. The concentration of each analyte was subsequently calculated using the regression equation associated with each drug.

#### Application to human plasma samples

Human plasma aliquots of 1 mL were seeded into a series of 10 mL volumetric flasks, each holding 3 mL of acetonitrile. Using suitable aliquots that encompassed the calibration ranges, all flasks were spiked with working solutions of APAP, NAPQI, and NAC. Employing a vortex shaker, the mixtures were well mixed prior to being centrifuged for 30 min. at a speed of 4000 rpm and the supernatant was evaporated under nitrogen.

##### HPTLC method

The residue was mixed with 0.1 ml of methanol again, and 10 μl were put on TLC plates. The overall technique described before was employed for estimating the concentration of APAP, NAPQI and NAC in human plasma samples.

##### HPLC method

The residue was mixed in 0.25 mls mobile phase and 20 μl injected into HPLC system. The overall technique described before was utilized to estimate the concentration of APAP, NAPQI and NAC in human plasma samples.

## Results and discussion

### Method optimization and development

#### HPTLC method

**Fig. 2:**
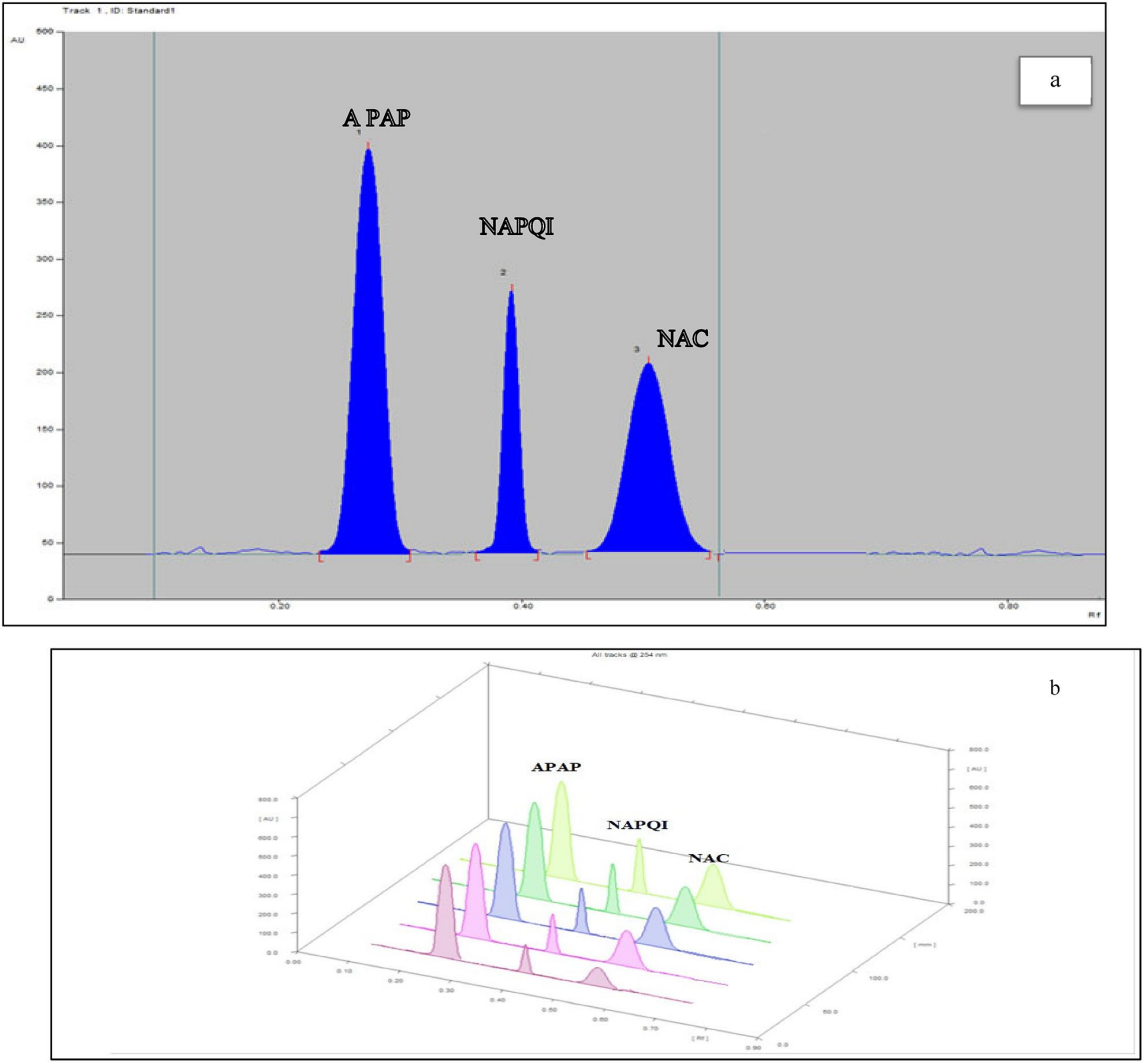
(**a**) 2 D HPTLC chromatogram of resolved mixture of 5 µg mL^−1^ APAP, 6 µg mL^−1^ NAPQI and 16 µg mL^−1^ NAC, (**b**) 3 D HPTLC chromatogram of resolved mixture of 5 µg mL^−1^ APAP, 6 µg mL^−1^ NAPQI and 16 µg mL^−1^ NAC.

Experiments employing various solvent systems, such as ethyl acetate + cloroform (4:4, 8:2, 9:1, 2:8, and 9.5:0.5, v/v), revealed inadequate separation of the compounds under investigation and the appearance of tailed peaks. Subsequently, minor amounts of toluene and triethylamine were added to ethyl acetate in an attempt to improve its performance, but no discernible improvement was observed. Improved spot formation was achieved by adding a small amount of acetic acid to ethyl acetate + methanol (8:2, v/v). Initial results demonstrated enhanced resolution and peak morphology. methanol: ethyl acetate: glacial acetic acid (8:2:0.2, v/v/v/v), which produced the most effective resolution of analytes and chromatographic neat separation (Fig. 2a, b) with R_f_-values of 0.25, 0.40, and 0.55 for APAP, NAPQI, and NAC, respectively. The densitometric measurements exhibited the greatest sensitivity at a wavelength of 254 nm, as determined through working across a range encompassing 220, 245, 265, and 315 nm. The sufficiency of the results obtained from estimating the system suitability parameters tailing factor, capacity factor, selectivity, retardation factor, and resolution)—to validate the performance of the enhanced chromatographic system^38^; the corresponding results are provided in Table 1.

**Table 1:** System suitability parameters of the two proposed HPTLC–densitometry and HPLC–DAD methods for determination of APAP along with NAPQI and NAC.

Method	Parameter	Reference values	APAP		NAPQI		NAC
HPTLC	Retardation factor (Rf)	–	0.25		0.40		0.55
	Capacity factor (k0)	1–10	1.01		1.52		2.34
	Selectivity(α)	≥ 1		2.04		1.48	
	Resolution (Rs)	> 1.5		1.60		2.78	
	Tailing factor(T)	≤ 2	0.99		1.32		1.44
Method	Parameter		APAP		NAPQI		NAC
HPLC	Capacity factor (k0)	1–10	2.34		5.48		7.08
	Selectivity(α)	≥ 1		1.89		3.45	
	Resolution (Rs)	> 1.5		4.43		6.25	
	Tailing factor(T)	≤ 2	1.45		1.56		1.27
	Column efficiency (N)	N > 2000	2358		4545		2747
	Height equivalent to theoretical plate(cm/plate)	The smaller the value the higher the column efficiency	0.0106		0.0055		0.0091
	Retention time tR (min)	–	2.435		3.812		5.093

#### HPLC method

**Fig. 3:**
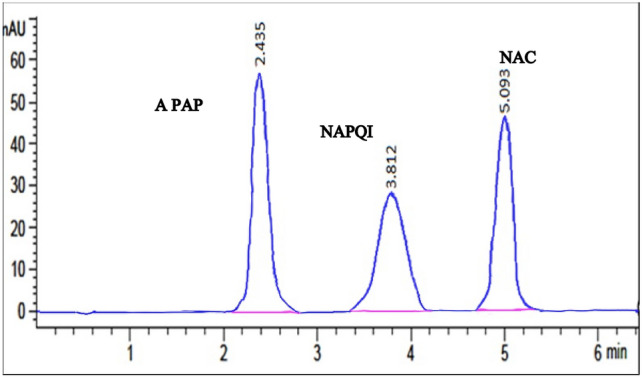
HPLC chromatogram of resolved mixture of 5 µg mL^−1^ APAP, 30 µg mL^−1^ NAPQI and 20 µg mL^−1^ NAC.

A quick, isocratic HPLC technique was devised. The method was developed and optimized using a Zorbax^®^ SB C_18_ analytical column (4.6 × 250 mm, 5 μm). Even though different mobile phases were tested, such as water–methanol (90:10, v/v), the separation that was achieved was not ideal. Diverse eluting systems, including water, methanol, and formic acid, were monitored in this study at varying flow rates, pH levels, and elution ratios. Tests showed that a mix of water, methanol, and formic acid, worked best for separation with little tailing. The amounts used were (70:30:0.15, v/v/v). When the flow rate was changed, it was found that a flow rate of 1 mL min^-1^ gave the best results for getting resolved peaks with the least amount of solvent use and run time. Finally, 254 nm was determined to be the optimal wavelength. As depicted in Fig. 3, the three analytes were effectively well separated in 5 min under these conditions: APAP, NAPQI, and NAC, with respective t_R_ values of 2.435, 3.812, and 5.093 min. Table 1 presents the outcomes pertaining to the parameters of system suitability, all of which fall within the permissible ranges^39^.

### Method validation

All validation items were adequately assessed to guarantee that the techniques met ICH demands^37^.

#### Linearity and range

In order to develop regression equations, calibration curves were first constructed to illustrate the relationship between peak area and component concentration. Table 2 illustrates overview of the regression parameters and ranges of linearity.

**Table 2:** Regression and validation parameters of the proposed HPTLC–densitometry and HPLC–DAD methods for determination of APAP along with NAPQI and NAC.

Method parameter	HPTLC-densitometry method			HPLC–DAD method		
	APAP	NAPQI	NAC	APAP	NAPQI	NAC
Range	0.5–6 μg/band	1–7 μg/band	0.3–18 μg/band	1–30 μg/mL	10–200 μg/mL	3–100 μg/mL
Slope	55.75	125.84	98.35	345.15	67.04	167.64
Intercept	− 43	− 34.22	12.19	− 280.09	− 123.37	70.92
Correlation coefficient (r)	0.9998	0.9999	0.9998	0.9999	0.9997	0.9998
Accuracy (mean ± SD)	100.43 ± 0.43	101.85 ± 1.35	100.87 ± 1.01	101.94 ± 1.29	100.99 ± 1.45	99.51 ± 1.22
LOD	0.126	0.211	0.083	0.314	2.659	0.558
LOQ	0.358	0.614	0.233	0.739	7.386	1.542
Precision (RSD%)						
Repeatability	0.843	0.357	0.454	0.893	0.593	0.845
Intermediate precision	1.393	1.427	1.945	1.468	1.359	1.375
Specificity (mean ± SD)	100.39 ± 0.956	101. 93 ± 0.780	99.12 ± 0.748	101.38 ± 1.836	101.83 ± 0.859	100.83 ± 0.943

#### Accuracy

The accuracy of the indicated methods was evaluated through analysis of APAP, NAPQI, and NAC using the prior chromatographic settings. Using the corresponding regression equation, the concentrations of each drug were calculated. Table 2 shows that the mean percent recoveries guaranteeing the correctness of the recommended methods were adequate.

#### Precision

To assess the intra- and inter-day precisions, assay of three chosen concentrations of each analyte were done three times on the same day and three times on consecutive days, respectively. Table 2 shows the results of calculating the percentage relative standard deviation.

#### Limits of detection and quantitation (LOD and LOQ)

The limits of detection and quantitation for APAP, NAPQI, and NAC were estimated employing the slope of the standard calibration curves and the residual standard deviations (Table 2). The low LOD and LOQ values obtained demonstrate the exceptional sensitivity of the offered techniques, allowing for sensitive investigation of analytes within the given limitations.

#### Robustness

A slight modification in the methanol ratio (± 1%), a refined adjustment in the distance travelled by the solvent (± 0.5 cm), and in the time of saturation (± 5 min) were elegantly employed to evaluate the robustness of the HPTLC method. Conversely, for the HPLC method, a variation in the scanning wavelength (± 1 nm) and a meticulously controlled alteration in the flow rate (± 0.1 mL/min) were impeccably utilized to appraise its robustness. The resulting range of values was perfectly appropriate. The opulent relative standard deviation (%) was found to be less than 2%, thus guaranteeing the utmost robustness of the procedures. Furthermore, the validation parameters remained firmly within the acceptable range, as elegantly displayed in Table S1 (Supplementary materials).

### Analysis of human plasma samples

Through the meticulous quantification of APAP, NAPQI, and NAC within the opulent confines of human plasma samples, the current two techniques have been exquisitely executed with resounding success Figures S1&S2 (Supplementary materials). Calibration curves was done in plasma and regression equations were developed for APAP, NAPQI and NAC, respectively as follows:

$${\text{Y}}\, = \,0.{\text{7543C}}\, + \,{1}.{545}.$$

$${\text{Y}}\, = \,0.{\text{432C}}\, + \,0.{243}.$$

$${\text{Y}}\, = \,0.{\text{254C}}\, + \,0.{466}.$$

These equations solved to unveil the exquisite concentration levels of each analyte, as magnificently displayed in the illustrious Table 3.

**Table 3:** Analysis of spiked human plasma samples containing APAP along with NAPQI and NAC the proposed HPTLC–densitometry and HPLC–DAD methods.

HPTLC-densitometry method						HPLC–DAD method					
APAP		NAPQI		NAC		APAP		NAPQI		NAC	
Added µg/band	%Recovery	Added µg/band	%Recovery	Added µg/band	%Recovery	Added µg/mL	%Recovery	Added µg/mL	%Recovery	Added µg/mL	%Recovery
1	102.23	1	103.09	1	107.46	1	112.43	15	109.53	5	105.44
2	106.23	4	105.73	4	103.87	5	106.66	30	105.87	20	108.88
3	112.34	5	109.34	10	104.73	20	104.65	100	109.76	70	110.53
5	110.34	6	102.85	16	103.89	25	103.89	150	104.97	90	103.97
Mean ± SD	107.78 ± 2.033	Mean ± SD	105.25 ± 2.256	Mean ± SD	104.98 ± 2.313	Mean ± SD	106.90 ± 2.117	Mean ± SD	107.53 ± 2.284	Mean ± SD	107.20 ± 2.493

### Evaluation of the greenness of the proposed technique

Studies have to consider into account the impact of the environment on research facilities in order to mitigate associated risks. The fundamental principles of “green chemistry” encompass the reduction of potentially hazardous solvents and reagents, the generation of less manageable waste, and the minimization of energy consumption. In the field of green chemistry (GC), significant emphasis is placed on the careful consideration of hazards related to various chemical and solvent ratios, types of samples, power consumption linked to complex equipment, and waste generated during sample preparation. Consequently, a variety of greenness standards were formulated to evaluate the environmental characteristics of the analytical methods^35,40–42^. The green attribute of the provided technique was assessed using two contemporary measures of greenness, namely GAPI and AGREE. The GAPI method was employed to assess the environmental characteristics of the process at each stage^36^. A pictogram is utilized to categorize the degree of greenness at various stages of an analytical process by employing a color scale that consists of either two or three levels of assessment. The AGREE technique is a method used to assess occupational and environmental risks related to the analytical process. It involves a comprehensive examination of 12 essential elements^35^. The output of the tool is a comprehensive analysis with insightful conclusions, represented as a value ranging from 0 to 1. A comparative analysis was conducted to assess the efficacy of the HPTLC method and the recommended HPLC methodology in identifying the level of greenness in the given method. The HPLC method offers three significant advantages over the HPTLC method, thereby establishing its superiority in terms of environmental friendliness. Initially, high-performance liquid chromatography (HPLC) necessitated a smaller volume of solvent compared to high-performance thin-layer chromatography (HPTLC). In addition, when compared to HPTLC, HPLC employs a reduced quantity of hazardous solvents. In summary, the high-performance liquid chromatography (HPLC) technique offers a higher level of sample analysis compared to high-performance thin-layer chromatography (HPTLC) due to its efficient separation time, as indicated in Table 4.

**Table 4:** Application of GAPI, AGREE and BAGI tools for the proposed HPTLC–densitometry and HPLC–DAD methods.

Method parameter	HPTLC-densitometry method			HPLC–DAD method			Reported method^26^		Reported method^19^	
	APAP	NAPQI	NAC	APAP	NAPQI	NAC	APAP	NAPQI	APAP	NAC
Range	0.5–6.0 μg/band	1.0–7.0 μg/band	0.3–18.0 μg/band	1.0–30.0 μg/mL	10.0–200.0 μg/mL	3.0–100.0 μg/mL	1–8 μg/mL	1–8 μg/mL	0.2–1000 μg/mL	0.02–100 μg/mL
Mobile Phase	Methanol: ethyl acetate: glacial acetic acid (8:2:0.2)			Water: methanol: formic acid (70: 30: 0.15)			Methanol, water, and buffer solution (20:40:40)		Gradient elution:10 mM aqueous ammonium acetate, pH 3.5 (A) and methanol (B)	
GAPI										
AGREE										
BAGI										

### Evaluation of the suggested techniques suitability

An opulent and extravagant platform for assessing the suitability of the current methodologies is the Blue Applicability Grade Index (BAGI). This tool evaluates ten essential factors, including the analysis category, sample productivity rate in analysis, analytical methodology, reagent quantities, required devices, simultaneous sample handling capability, preconcentration demand, level of process automation, sample construction method, sample quantity, and the ability to estimate analytes concurrently. The color scheme used to represent various levels of acceptance is as follows: white denotes lack of acceptance, light blue indicates minimal acceptance, blue signifies reasonable acceptance, and dark blue represents optimal acceptance^43,44^. In order to meet the criteria of being deemed "acceptable," the method must achieve a minimum score of 60 points. The level of applicability of the approach is demonstrated by the BAGI rating awarded, which is 80 for the HPTLC method and 82.5 for HPLC and the reported methods, as shown in Table 4.

## Limitations and future opportunities

The HPLC–DAD approach was not evaluated in animal tissues, and our primary purpose was to analyze analytes in biological fluid using a simple and accessible chromatographic apparatus. Furthermore, the LC–MS technology might be the optimum one for analyte evaluation in fluids and animal tissues since it allows for detection at the nanogram amount. Furthermore, various detectors, such as the evaporative light scattering detector (ELSD), refractive index, and corona spray, should be investigated to increase sensitivity.

## Conclusion

In this work, HPTLC and HPLC methods have been created in order to determine; APAP, NAPQI, and NAC in human plasma samples, which is a great chance to measure drugs, metabolites, and antidotes in one analytical run on a green basis. Given the opulence of the established HPLC procedure, a mere modicum of solvents is required for the complete separation, so it is very cost-effective and exhibiting exceptional efficiency. The highly esteemed HPTLC method upholds the utmost accuracy, unparalleled sensitivity, exquisite selectivity, and impeccable precision in the realm of analytical determination. It is also a good, safe method that could lead to improvements in how little sample preparation is needed, how little solvent is used, how quickly the analysis is done, and how much it costs. Our motivation to develop green analysis-based methods stemmed from the increasing recognition of the detrimental effects that solvents and chemicals have on the environment.

## Supplementary Information

Supplementary Information.

## Data Availability

All data generated or analysed during this study are included in this published article.
