# Supplementary material for: Two chromatographic methods for analyzing paracetamol in spiked human plasma with its toxic metabolite, N-acetyl parabenzoquinone imine and its antidote, N-acetyl-l-cysteine
Source: Sci Rep. 2025 Feb 3;15:4119. doi: 10.1038/s41598-025-86070-3 (PMC11791058; doi:10.1038/s41598-025-86070-3)
Supplement: Supplementary file 1 — Supplementary Information. [file 41598_2025_86070_MOESM1_ESM.docx]

**Two Chromatographic Methods for Analyzing Paracetamol in Spiked Human Plasma with N-acetyl parabenzoquinone Imine and N-acetyl-L-cysteine**

**Omar M El-Abassy, Michael Gamal Fawzy, Ebraam B. Kamel**


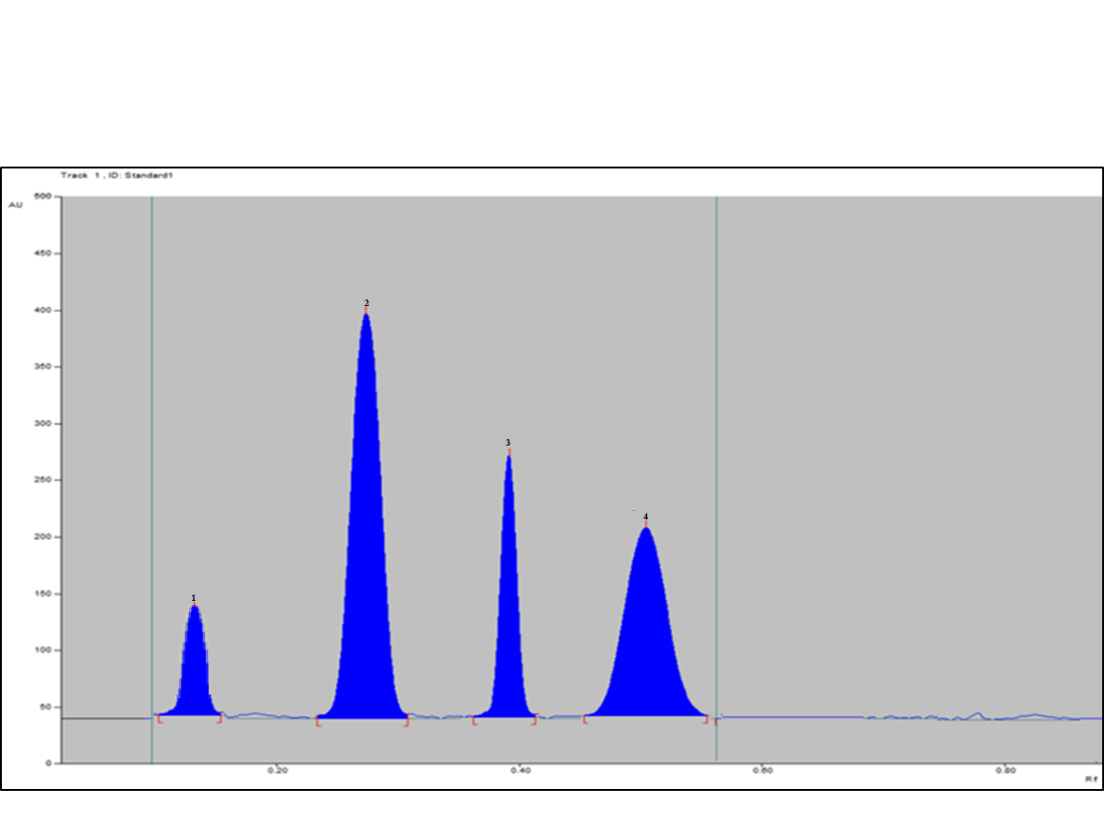


Plasma peak

Figure S1: 2 D HPTLC chromatogram of mixture of 5 µg mL^-1^ APAP, 6 µg mL^-1^ NAPQI and 16 µg mL^-1^ NAC resolved from plasma peak


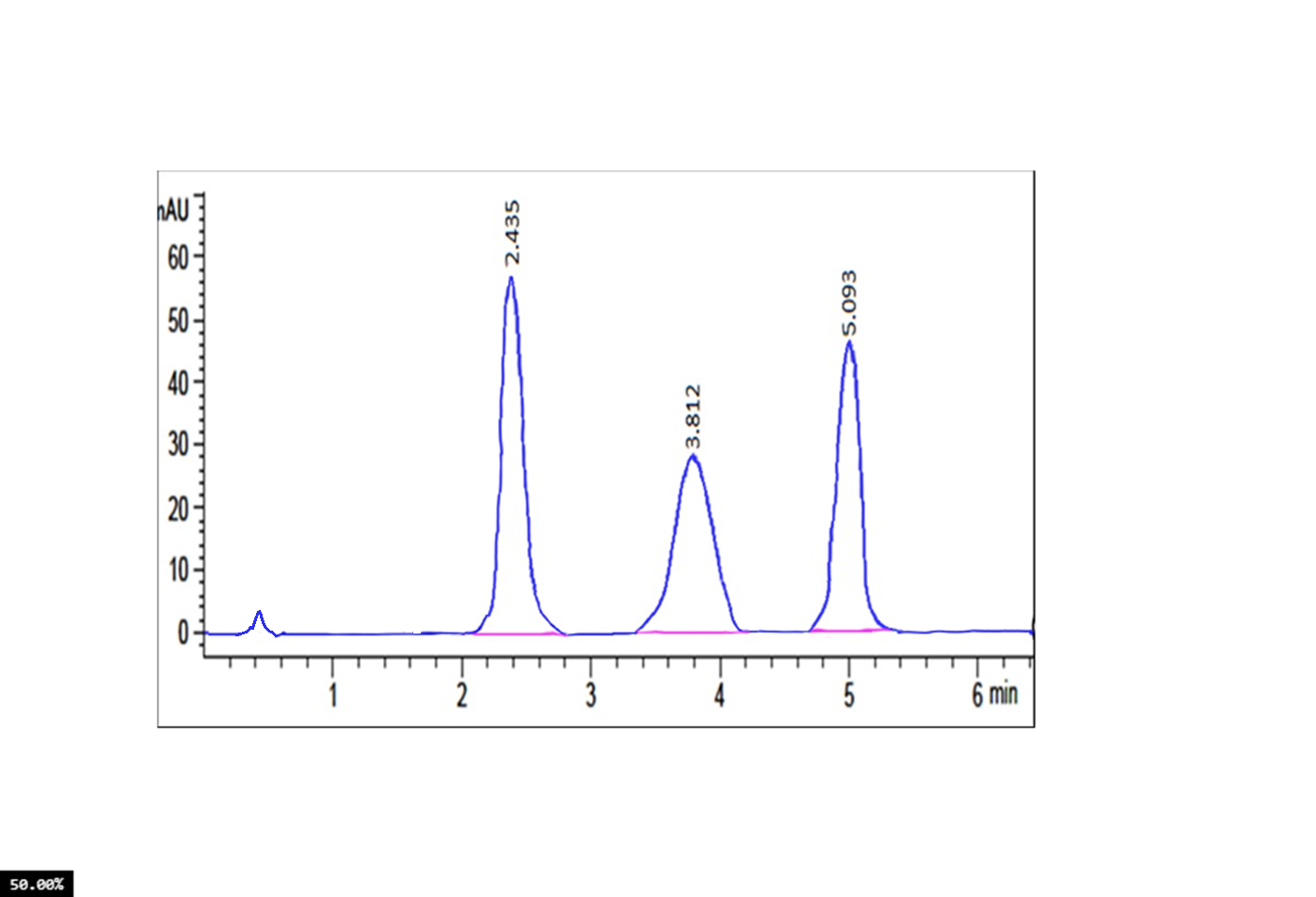


Plasma peak

Figure S2: HPLC chromatogram of mixture of 5 µg mL^-1^ APAP, 30 µg mL^-1^ NAPQI and 20 µg mL^-1^ NAC resolved from plasma peak

**Table S1:** Robustness results of the proposed HPLC and HPTLC methods

| **(%RSD) ^a^** | | | | | | | **Parameter** |
| --- | --- | --- | --- | --- | --- | --- | --- |
| **HPLC** | | |  | **HPTLC** | | |  |
|  |  |  |  |  |  |  |  |
| 1.723 | 0.453 | 1.432 | **Scanning wavelength (±1 nm)** | 0.143 | 1.734 | 0.764 | **Methanol ratio (±1%)** |
| 0.953 | 0.674 | 0.578 | **Flow rate (±0.1 mL/min)** | 1.232 | 0.863 | 0.945 | **Distance travelled by the solvent (±0.5 cm)** |
|  |  |  |  | 1.344 | 1.855 | 1.355 | **Time of saturation (±5 min)** |
